# Multifunctional OEO‐ZIF‐8‐HA Nanoparticles for Antibacterial Control on Latex Surfaces and Baby Arugula (*Eruca Sativa*) Leaves

**DOI:** 10.1111/1750-3841.70896

**Published:** 2026-02-06

**Authors:** Huy Loc Nguyen, Rosana G. Moreira, M. Elena Castell‐Perez

**Affiliations:** ^1^ Department of Biological and Agricultural Engineering Texas A&M University College Station Texas USA

**Keywords:** biofilms, food safety, HA, nanotechnology, OEOs, ZIF‐8

## Abstract

Persistent contamination of food‐contact surfaces and leafy greens by *Listeria monocytogenes* continues to challenge fresh‐produce safety, particularly as the industry seeks effective alternatives to chlorine‐based sanitizers. To address this need, this study developed and evaluated multifunctional antimicrobial nanocomposites composed of oregano essential oil (OEO), hyaluronic acid (HA), and metal‐doped (Ag and Fe) zeolitic imidazolate framework‐8 (ZIF‐8). Three formulations, OEO‐Ag‐ZIF‐8‐HA, OEO‐Fe‐ZIF‐8‐HA, and OEO‐HA, were synthesized and assessed for their antimicrobial performance on latex food‐contact surfaces and a common leafy green found in ready‐to‐eat salads, baby arugula (*Eruca sativa*) leaves. OEO‐Ag‐ZIF‐8‐HA demonstrated the strongest antibacterial activity, achieving >5‐log reductions on latex surfaces and 3.39‐log CFU/g reductions in 15 min on arugula, surpassing the efficacy of a 200‐ppm chlorine control. Cytotoxicity testing indicated low cytotoxicity under the tested conditions (>85% cell viability), with the metal‐free OEO‐HA formulation showing the most favorable response at concentrations below 500 µg/mL. Quality evaluations further confirmed that the treated arugula retained color, freshness, and firmness during five days of storage at 21°C. Collectively, these findings highlight the potential of OEO‐based, HA‐functionalized, and metal‐doped ZIF‐8 nanocomposites as promising chlorine‐free antimicrobial candidates for improving the sanitation of food‐contact surfaces and enhancing the microbial safety of leafy greens.

**Practical Applications:**

The developed nanocomposites offer a safe, chlorine‐free antimicrobial alternative for controlling *Listeria monocytogenes* and other pathogens on food‐contact surfaces and fresh produce such as baby arugula (*Eruca sativa*) leaves.

## 1 Introduction

Foodborne illnesses remain a critical public health and economic concern worldwide, with *Listeria monocytogenes* (*L. monocytogenes*) among the most persistent and life‐threatening pathogens in the food industry (Arthur and Gil 2025). Listeriosis, the disease caused by *L. monocytogenes*, is associated with high hospitalization and mortality rates, particularly among vulnerable populations (Leong et al. 2014; Osek and Wieczorek 2022). In the past decade, *L. monocytogenes* contamination of fresh produce has led to several high‐profile outbreaks and recalls. For instance, in 2015–2016 a multistate listeriosis outbreak linked to packaged salad greens resulted in 19 cases (including one death) and triggered the recall of 22 salad products (Self et al. 2019). Recently, in 2021, an Ohio farm recalled a variety of greenhouse‐grown leafy greens, including arugula, after routine testing detected *L. monocytogenes* (FDA 2021). Such incidents underscore the vulnerability of raw, ready‐to‐eat leafy vegetables to *L. monocytogenes* contamination and reinforce the rationale for using baby arugula as a test matrix for novel antimicrobial strategies.

Unlike other bacteria, *L. monocytogenes* tolerates refrigeration, high salinity, and mild acidity, complicating its eradication from food‐processing facilities (Skowron et al. 2018; Klopper et al. 2023). *Listeria monocytogenes* strains ATCC 19115, ATCC 35152, and ATCC 7644 are well‐characterized isolates with diverse origins and important roles in food safety research. ATCC 19115 (serotype 4b) is a human clinical isolate from cerebrospinal fluid and represents a highly virulent lineage often implicated in listeriosis outbreaks (Wen et al. 2009; Zurawik et al. 2024). It is frequently used as a positive control in biofilm formation assays and antimicrobial efficacy tests. ATCC 35152 (NCTC 7973, serotype 1/2a) was originally isolated from a Guinea pig's mesenteric lymph node and is commonly found in food environments (Zurawik et al. 2024; UK Health Security Agency 2025). It is widely used as a representative food‐related strain in biofilm studies and as a quality control organism in *Listeria* testing methods (Seres‐Steinbach et al. 2025). ATCC 7644 (“Gibson”) is a fully sequenced human isolate that is routinely employed as a challenge strain in ready‐to‐eat food studies and as a reference culture in laboratory assays (e.g., growth potential and pathogen detection validations) (Skalina and Nikolajeva 2010). While not tied to any specific outbreak, all three strains are standard choices in challenge trials, antimicrobial testing, and regulatory validation studies owing to their reproducible biofilm‐forming ability and well‐documented virulence profiles (Simonavičienė et al. 2021).

Their persistence is due to robust biofilm formation on diverse food‐contact materials, including stainless steel, polyurethane, polyethylene, and latex (Olanbiwoninu and Popoola 2023; El‐Sawy et al. 2024). These biofilms function as reservoirs for recurrent contamination, shedding planktonic cells and resisting standard sanitation (Rosario et al. 2024; Coppola et al. 2025). Latex components in gloves and seals are particularly prone to microbial adhesion due to hydrophobicity and microtopography (Mazaheri et al. 2021).

Biofilms display strong resistance to disinfectants through their extracellular polymeric matrix and altered metabolism (Mirghani et al. 2022). Conventional sanitizers (chlorine, quaternary ammonium, and peroxyacetic acid) often lose efficacy against mature biofilms and may corrode equipment or form toxic residues (FAO 2008; Mendoza et al. 2022). Consequently, innovative, non‐toxic antimicrobial strategies are being explored (Grooters et al. 2024; Sevimli‐Yurttas et al. 2024). Nanotechnology provides an effective platform for biofilm control by combining multiple antibacterial mechanisms within a single nanostructure. EO‐loaded nanoparticles, especially those incorporating OEO, are promising due to the potent phenolic compounds carvacrol and thymol, which disrupt bacterial membranes and induce leakage of intracellular contents (Luo et al. 2022; Zamuner et al. 2023). OEO has demonstrated strong antibiofilm efficacy, though its volatility and hydrophobicity limit stability and persistence (Li et al. 2022; Knežević et al. 2023). Encapsulation of essential oils within nanoparticles improves dispersibility, protects volatile actives, and allows controlled release (Sharma et al. 2022).

Metal–organic frameworks (MOFs), particularly ZIF‐8, have emerged as ideal EO carriers owing to their high surface area, tunable porosity, and pH‐responsive degradation (Liu et al. 2019b; Sadeq et al. 2025). ZIF‐8 releases Zn^2^^+^ ions that exhibit intrinsic antibacterial activity (Wei et al. 2022). Doping ZIF‐8 with metals such as Ag or Fe enhances its antimicrobial potency via synergistic ion release and reactive oxygen species (ROS) generation (Ximing et al. 2017; Nguyen 2025). These composites penetrate biofilms, disrupt cellular integrity, and outperform conventional sanitizers (Omran et al. 2024). Furthermore, doped ZIF‐8 frameworks can co‐deliver natural antimicrobials for enhanced, sustained effects (Li et al. 2023). To improve dispersion and biocompatibility, nanoparticles can be coated with HA, a naturally derived, non‐toxic polysaccharide known for its hydrophilicity, stability, and anti‐adhesive properties (Iaconisi et al. 2023). Although the antimicrobial efficacy of HA‐containing nanoparticles was evaluated under laboratory conditions in this study, with MW = 1500 kDa, HA can be produced in food‐grade form via microbial fermentation and is commercially available for food and dietary applications (Salih et al. 2024). This indicates that, following appropriate regulatory validation, the proposed HA‐based nanoparticle systems have potential applicability in food contact and fresh produce processing environments (Xia et al., 2019). HA coatings prevent initial bacterial adhesion and stabilize nanoparticles in aqueous systems (Tan et al. 2023; Ye et al. 2025). Although HA alone is not bactericidal (Drago et al. 2014), combining it with metal‐doped ZIF‐8 and OEO creates a multifunctional system that integrates antiadhesion and antibacterial mechanisms (Paris et al. 2019).

Arugula (*Eruca sativa*), like other leafy greens, is highly prone to microbial spoilage due to its moisture‐rich microstructure (Komeroski et al. 2024; Zhu et al. 2025). Its leaves have uneven surfaces with folds and tiny pockets where bacteria can hide, making it easy for Listeria to stick and even form protective layers, which is why rinsing alone often is not enough to get rid of it. Metal‐doped ZIF‐8 frameworks (Fe and Ag) were chosen in this research due to their ability to enhance ion release and generate ROS, thereby amplifying antibacterial efficacy against resilient pathogens. While essential oils have been encapsulated in various nanoparticle systems for antimicrobial applications (Asensio et al. 2020; Chiriac et al. 2021), the present work advances the field through a distinctive formulation and application. In contrast to prior approaches, we incorporated OEO into a ZIF‐8 MOF scaffold doped with Ag/Fe and coated with HA, yielding a single nanocomposite that provides controlled OEO release, concurrent antimicrobial ion delivery (Zn^2^^+^ from ZIF‐8 and Ag^+^/Fe^2^^+^ from Ag‐ZIF‐8), and anti‐adhesive surface properties from the HA layer (Tahric et al. 2023). This combination of functionalities has not been previously reported and represents a novel strategy to enhance the efficacy and stability of EO‐based antimicrobials. Moreover, by applying these OEO–ZIF‐8–HA nanocomposites to both food‐contact surfaces (latex) and actual produce (baby arugula leaves), our study demonstrates a practical, chlorine‐free intervention for *L. monocytogenes* control, bridging laboratory efficacy with real‐world conditions.

Therefore, this study aimed to develop and evaluate multifunctional nanoparticles composed of OEO, HA, and metal‐doped ZIF‐8 (Ag and Fe) for controlling *L. monocytogenes* on latex food‐contact surfaces and baby arugula (*Eruca sativa*) leaves with the specific objectives included: (i) synthesizing and characterizing OEO‐Ag‐ZIF‐8‐HA, OEO‐Fe‐ZIF‐8‐HA, and OEO‐HA nanoparticles; (ii) sasessing nanoparticles' antibacterial efficacy against *L. monocytogenes* through several microbial and biofilm inhibition assays; (iii) evaluating the application to leafy greens processing and their impact on microbial reduction and quality preservation; and (iv) Comparing nanoparticles’ performance with conventional chlorine treatments.

## 2 Materials and Methods

### 2.1 Nanoparticle Synthesis

The synthesis of the antimicrobial nanocomposite systems involved a sequential, multi‐step fabrication process encompassing the preparation of metal‐doped ZIF‐8 nanoparticles, HA surface functionalization, essential‐oil loading, and HA‐based nano‐encapsulation. Ag‐ZIF‐8 was synthesized following Nguyen et al. (2025) with slight modifications by combining zinc nitrate hexahydrate (1.96 mmol), silver nitrate (0.84 mmol), and 2‐methylimidazole (64.4 mmol) in ethanol (1.4 × 10^3^ mmol) within Erlenmeyer flasks, followed by the rapid addition of the ligand solution into the metal precursor mixture under constant stirring at 21°C for 24 h to promote metal–ligand coordination and crystal growth. The resulting precipitates were isolated by centrifugation (8000 rpm, 10 min), washed three times with ethanol to remove unreacted components, and vacuum‐dried before storage in a desiccator. Fe‐ZIF‐8 was produced identically, substituting silver nitrate with iron sulfate heptahydrate. To generate HA‐functionalized metal‐doped nanoparticles (Ag‐ZIF‐8‐HA and Fe‐ZIF‐8‐HA), the dried ZIF‐8 powders were dispersed into a 0.05 mg/mL HA (MW = 1500 kDa) aqueous solution, which was used in this study as a functional biopolymer for antimicrobial performance evaluation under laboratory conditions, at a 1:2 mass‐to‐volume ratio, vortexed for 2 min for complete wetting, stirred for 30 min to allow electrostatic adsorption of HA onto the positively charged ZIF‐8 surface, sonicated for 1 min to ensure uniform coating, and centrifuged (8000 rpm, 10 min) to remove excess HA. The pellet was washed thrice with DI water and vacuum‐dried for 4 h to obtain stable HA‐coated powders. Essential‐oil–loaded variants (OEO‐Ag‐ZIF‐8‐HA and OEO‐Fe‐ZIF‐8‐HA) were synthesized by dispersing 0.5 g of the HA‐coated nanoparticles into 10 mL ethanol, followed by a drop wise addition of OEO (100 drops) to facilitate hydrophobic–hydrophilic interactions and diffusion‐driven loading into the ZIF‐8 porous framework. The mixtures were stirred for 30 min, sonicated for 1 min to enhance penetration of OEO into the hierarchical pores and HA outer layer, centrifuged (8000 rpm, 10 min), washed three times with DI water to remove unbound OEO, and vacuum‐dried for 4 h prior to desiccator storage at 21°C. Parallel synthesis using Fe‐ZIF‐8‐HA yielded OEO‐Fe‐ZIF‐8‐HA with identical processing parameters.

Independently, an HA‐only encapsulated essential‐oil system (OEO‐HA) was produced by dissolving HA in DI water (1:3 mg/mL) for 6 h, preparing an OEO–ethanol phase (1:3 mL/mL) emulsified with Tween 80 to improve miscibility, and introducing the oil phase into the HA solution under 5 min of sonication to promote droplet size reduction and encapsulation. The suspension was stirred for an additional 6 h to stabilize HA–OEO interactions, then vacuum‐dried at 35°C to gently remove solvent while preventing volatilization of bioactive oil constituents. The concentrated samples were subsequently freeze‐dried at −80°C for 72 h to obtain dry, free‐flowing OEO–HA powders suitable for downstream characterization and antimicrobial testing.

### 2.2 Nanoparticle Characterization

Briefly, nanoparticles were characterized by morphology (scanning electron microscopy (SEM), transmission electron microscopy (TEM)), elemental distribution (EDS mapping), functional groups Fourier transform infrared (FTIR), crystallinity X‐ray diffraction (XRD), surface charge (zeta potential (ZP)), and particle size (PS) (DLS) (Jongert et al. 2024; Nguyen 2025). SEM and TEM imaging confirmed nanoscale dimensions and surface uniformity. and XRD verified structural integrity and successful incorporation of OEO and metal dopants, while ZP and DLS analyses assessed colloidal stability and dispersion behavior.

Morphological analysis was performed using SEM (Quanta 600 FEG SEM, Texas A&M University, College Station, TX, USA) after sputter‐coating samples with a thin platinum layer to enhance conductivity. SEM imaging, conducted at 20 kV with magnifications of 2000×–4000×, provided detailed surface morphology, while energy‐dispersive X‐ray spectroscopy (EDS) coupled with SEM enabled elemental mapping (MAP), generating color‐coded distributions of key elements and confirming uniform elemental incorporation across nanoparticle surfaces. TEM was further employed to resolve nanoscale structural features. Nanoparticles were dispersed in ethanol, sonicated, drop‐cast onto carbon‐coated copper grids, and imaged using a JEOL 1200 EX microscope operating at 200 kV. For each formulation, at least ten fields of view were examined at magnifications ranging from 50,000× to 200,000× to ensure representative analysis of PS, shape, and dispersion, with optional uranyl acetate staining applied to enhance contrast.

Chemical integrity and functional group composition were verified by FTIR spectroscopy (IR Prestige‐21 Spectrometer, Texas A and M University, College Station, TX, USA) in attenuated total reflectance mode over a spectral range of 400–4000 cm^−^^1^, with measurements performed in triplicate to ensure reproducibility. Crystallographic structure was assessed by XRD using a Bruker D8 Endeavor diffractometer with Cu Kα radiation, following standardized protocols to identify characteristic diffraction peaks and confirm retention of crystalline ZIF‐8 frameworks after HA coating and OEO loading.

Surface charge and colloidal stability were evaluated via ZP measurements using a Malvern Zetasizer Nano ZS, with nanoparticles dispersed in ethanol (2 mg/mL), sonicated, and analyzed at 25°C in triplicate. PS distribution was measured by dynamic light scattering under similar dispersion conditions, providing insight into hydrodynamic diameter and aggregation behavior. Finally, thermal stability and phase transitions were examined using differential scanning calorimetry (DSC), where sealed nanoparticle samples were heated from 20 to 400°C under a nitrogen atmosphere. DSC thermograms allowed identification of endothermic and exothermic events associated with melting, decomposition, or structural transitions.

### 2.3 Cytotoxicity Assay

Cytotoxicity was evaluated using the Cell Counting Kit‐8 (CCK‐8; CP002, Signalway Antibody, MD, USA) assay. Nanoparticle suspensions were incubated with mammalian cells for 48 h, and cell viability was measured via absorbance at 450 nm. Concentrations up to 2000 µg/mL were tested to determine dose‐dependent effects (Bai et al. 2021).

### 2.4 Antibacterial Testing

Antibacterial efficacy against a mixture of several *Listeria monocytogenes* strains (ATCC 19115, 35152, 7644) was assessed using disk diffusion, minimum inhibitory concentration (MIC), and minimum bactericidal concentration (MBC) assays. Frozen stocks (–80°C) of each strain were obtained from the Microbiology Laboratory culture collection at Texas A and M University. For preparation of a multi‐strain cocktail, overnight cultures of each strain were adjusted to approximately 10^8^ CFU/mL. Equal volumes (1 mL) of each suspension were combined in sterile TSB, vortexed for 2 min to ensure homogeneity, and used immediately for downstream assays to simulate multi‐strain contamination scenarios (Yehia et al. 2016).

For the disk diffusion test, the synthesized nanoparticle powders were dispersed in deionized water at a concentration of 1500 µg/mL and 0.2 mL of each suspension was loaded onto sterile filter paper disks (0.5 cm diameter; Whatman, Sigma‐Aldrich, MO, USA) and placed on the inoculated TSA plates (at a 0.5 cm depth) incubated at 37°C for 24 h, after which the diameters of inhibition zones were measured in millimeters (Hara et al. 2023). The nanoparticle ratio exhibiting the largest inhibition zone was selected for further antibacterial optimization. MIC and MBC values were determined via broth microdilution in 96‐well plates, with OD_630_ readings taken hourly over 24 h of incubation. MBC was defined as the lowest concentration that yielded a ≥3‐log CFU reduction on TSA plates (Nguyen 2025). All experiments were performed in triplicate to ensure reproducibility, with untreated inoculum and media‐only wells serving as positive and negative controls, respectively.

### 2.5 Biofilm Inhibition on Latex Surfaces

#### 2.5.1 Surface Preparation

Latex coupons (1 cm × 1 cm × 0.008 cm) were initially cleaned using Liquinox (Alconox Inc., NY, USA), thoroughly rinsed with distilled water, and dried at 60°C. After washing and water rinsing, they were treated with a 500‐ppm chlorine solution for 30 min, dried overnight in a biosafety cabinet (Labconco, MO, USA), and sterilized by UV irradiation on both surfaces for 30 min. The prepared coupons were then stored aseptically in sterile tubes at room temperature (21°C) for further use (Nguyen 2025).

#### 2.5.2 Surface Contact Angle

The wettability of the latex surfaces was determined using the Pendant Drop Goniometer OCA 11 (DataPhysics Instruments, Stuttgart, Germany) by measuring the contact angles of both water and nanoparticle suspensions (1000 µg/mL concentration). For each measurement, a 6 µL droplet was carefully deposited onto the material surface, and digital images were immediately recorded (Arcot et al. 2021). Static contact angles were quantified using ImageJ software (National Institutes of Health, MD, USA). All measurements were performed at 21°C, and the values reported represent the average of three independent replicates.

#### 2.5.3 Surface Tension

The surface tension of the nanoparticle dispersions was measured with a dynamic contact analyzer (DCA‐315, Cahn Scientific, Irvine, CA, USA). Each dispersion was prepared at the target concentrations (250, 500, 1000, and 2000 µg/mL) in DI water, gently mixed to ensure homogeneity before testing. For each sample, the force as a platinum plate was immersed at a constant rate recorded, and the equilibrium surface tension (mN/m) was computed from the force–wetting geometry (Chen and Wu 2011). Measurements were performed in triplicate per formulation.

#### 2.5.4 Biofilm Formation

For inoculum preparation, a loopful of working stock was transferred into 9 mL of TSB and incubated at 37°C for 24 h. Following incubation, the culture was centrifuged (6000 rpm, 10 min, 21°C) and washed three times with sterile 0.1% peptone water (PW). The pellet was resuspended in TSB, and the optical density was measured at 600 nm (OD_600_). The bacterial suspension was adjusted to 10^8^ CFU/mL using a pre‐established OD–CFU calibration curve, and the concentration was confirmed by spread‐plating serial dilutions on TSA. Ten latex coupons (1 cm × 1 cm) were prepared as described in 2.5.1, sterilized, and placed in sterile 100 mm petri dishes. 30 mL of bacterial suspension (10^8^ CFU/mL in TSB) was added to each dish to submerge the coupons fully. Samples were incubated statically at 37°C for 72 h to promote biofilm development. To facilitate the formation of mature, surface‐attached biofilms rather than transient cell attachment, 50% of the spent medium was gently replaced with fresh sterile TSB every 24 h without disturbing the coupons, ensuring sustained nutrient availability while preserving established extracellular polymeric substance (EPS) matrices (Harvey et al. 2007). This extended incubation and nutrient supplementation are necessary for the development of mature biofilms (Nguyen 2025).

#### 2.5.5 Effect of Nanoparticle Solutions on Biofilm Formation on Latex Surfaces

The bacterial inoculum was prepared as described in Section 2.5.4, with *L. monocytogenes* (∼10^8^ CFU/mL) exposed to OEO–Fe–ZIF‐8–HA, OEO–Ag–ZIF‐8–HA, or OEO–HA nanoparticles dispersed in PBS containing 5% MeOH. Nanoparticle concentrations were adjusted to the MIC, MBC, and 2 × MBC levels. Sterile coupons were immersed in these suspensions and incubated for 72 h at 37°C to promote biofilm formation.

After incubation, coupons were aseptically removed and rinsed three times with 3 mL sterile water to eliminate planktonic and loosely attached cells, ensuring that only firmly attached biofilm‐associated cells remained on the surface. The effectiveness of this rinsing step in removing non‐biofilm cells has been well established in surface‐associated biofilm studies (Choi et al. 2012). Coupons were then transferred into sterile centrifuge tubes containing 1 g of sterile glass beads (500 µm; BioSpec Products, OK, USA) and 5 mL of 0.1% PW. Biofilm cells were mechanically detached by vortexing (Vortex‐Genie 2, Scientific Industries, PA, USA) for 1 min, a procedure shown to disrupt EPS matrices and release biofilm‐embedded bacteria without significant loss of viability.

The resulting suspensions were serially diluted in buffered peptone water (Difco, NJ, USA) and incubated aerobically at 37°C for 72 h for colony enumeration. All treatments were conducted in triplicate, with PBS + *L. monocytogenes* and PBS + 5% MeOH serving as positive and solvent controls, respectively (Sevimli‐Yurttas 2024). The combination of extended incubation, repeated rinsing, and mechanical disruption ensured that recovered cells represented mature biofilm populations rather than loosely attached or planktonic bacteria.

#### 2.5.6 Effect of Nanoparticle Solutions on the Established Biofilms

*L. monocytogenes* biofilms were incubated for 72 h, after which coupons were aseptically retrieved and rinsed three times with 3 mL sterile water to remove loosely attached cells. Coupons were then treated with nanoparticle solutions (prepared in PBS + 5% MeOH) for either 1 h or 24 h under static conditions. To simulate mechanical cleaning, a “scrubbing” treatment was also tested by vortexing coupons in 2 mL nanoparticle solution containing 100 mg sterile glass beads (500 µm, BioSpec Products, OK, USA) at 100 rpm for 1 min, followed by static incubation for 1 h. Controls were prepared identically using PBS + 5% MeOH.

After treatment, coupons were rinsed thrice with sterile water, placed in tubes with 1 g of sterile glass beads and 5 mL of 0.1% peptone water, and vortexed 1 min (Vortex‐Genie 2, Scientific Industries, PA, USA) to detach biofilm cells. Aliquots (1 mL) were serially diluted in 0.1% PW, and 0.1 mL of suitable dilutions were spread on TSA plates for enumeration. Plates were incubated at 37°C for 24 h. The 1‐h treatment was performed in triplicate on two separate days, and the 24‐h treatment in three replicates (Sevimli‐Yurttas 2024).

### 2.6 OEO‐HA Nanoparticle Applications to the Baby Arugula Leaves’ Surfaces

Fresh baby arugula (Kroger, Bryan, TX, USA) was stored at 4°C and used within 24 h. Damaged or wilted leaves were removed. The leaves were inoculated with the *L. monocytogenes* cocktail (10^8^ CFU/mL) and dipped for 1‐, 2‐, 5‐, and 10‐min in (1) OEO‐Fe‐ZIF‐8‐HA at 875 µg/mL; (2) OEO‐Ag‐ZIF‐8‐HA at 125 µg/mL; and (3) OEO‐HA at 1250 µg/mL nanoparticle solutions. Previous optimization studies conducted in our laboratory showed that dipping treatments were superior to spraying for removing bacteria from a surface (Yang et al. 2022; Nguyen et al. 2025). Microbial reductions were quantified using Oxford Listeria Agar. A 200‐ppm chlorine solution, formulated by diluting commercial sodium hypochlorite (7.4% Clorox bleach, Clorox Co., CA, USA) in deionized water, served as the chemical sanitizing control, while sterile distilled water served as the negative control at the exact exposure durations. Chlorine concentration was verified using chlorine test strips (Micro Essential Laboratory, Brooklyn, NY, USA) by immersing the strips in the chlorine solution, then removing and comparing them to the color reference chart. Residual free chlorine concentrations were measured after contact with the leafy greens using chlorine test strips. In the study, chlorine test strips were employed to rapidly verify free chlorine levels immediately after treatment, as chlorine decays quickly in the presence of organic matter on leafy greens. Residual free chlorine concentrations fell below the detectable range of the test strips (<10 ppm) following contact with the leaves. The chlorine solution was adjusted until the color matched the 200‐ppm chlorine reference level. Leaves were then air‐dried for 15 min on each side under a biosafety cabinet, portioned into 10 g samples, and transferred into sterile Whirl‐Pak stand‐up sample bags (Nasco Education, WI, USA). Each sample was mixed with 90 mL of sterile 0.1% (w/v) peptone water and manually massaged to detach surface bacteria. The reduction of viable bacteria was calculated as the difference in log CFU between untreated controls and treated samples. All tests were performed in triplicate, with duplicate plating for each repeat to guarantee reproducibility.

Quality attributes of the arugula leaves, including visual appearance and color, were evaluated on the day of sample preparation (day 0) and on days 1, 3, and 5 post‐sample preparation at 21°C (Ly et al. 2020; Nguyen et al. 2025). Color changes in baby arugula leaves were evaluated using a LabScan XE 16437 colorimeter (HunterLab Inc., VA, USA) operated with Universal Software version 3.80. The instrument was calibrated prior to analysis with standard black and white reference tiles. Measurements were performed at 21°C, with three readings taken per sample to ensure accuracy. Results were expressed in the CIE Lab* color space (Cabahug et al. 2019).

Although OEO is generally recognized for its antimicrobial efficacy, its strong aroma may influence sensory perception at elevated concentrations. While no visible quality deterioration was observed in treated arugula, formal sensory evaluation was not conducted in this study. Future work should include sensory analysis to determine consumer acceptability thresholds and optimize formulation concentrations for commercial application.

### 2.7 Statistical Analysis

All experiments were conducted using a completely randomized design with three independent biological replicates for each treatment. Each biological replicate consisted of separately prepared samples processed on different days. For microbiological assays, each replicate was plated in duplicate, and colony counts were averaged prior to statistical analysis. Physicochemical and cytotoxicity measurements were performed with three independent measurements per formulation, and results are reported as means ± standard deviations (SD). Statistical analyses were carried out using JMP Pro 17 software (SAS Institute, Cary, NC, USA). Treatment effects were evaluated using one‐way analysis of variance (ANOVA), followed by pairwise comparisons with Student's t‐test when significant differences were detected, with statistical significance defined at *p* < 0.05.

## 3 Results and Discussion

### 3.1 Nanoparticle Characterization

#### 3.1.1 SEM With MAP

**FIGURE 1:**
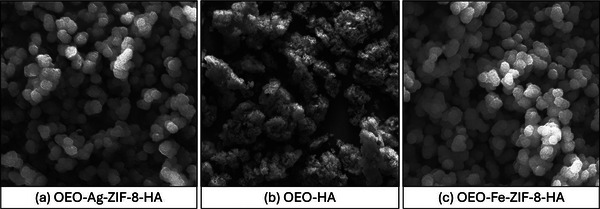
SEM pictures of (a) OEO‐Ag‐ZIF‐8‐HA; (b) OEO‐HA; and (c) OEO‐Fe‐ZIF‐8‐HA nanoparticles with magnification levels ranging from 1000 to 5000*x*.

**FIGURE 2:**
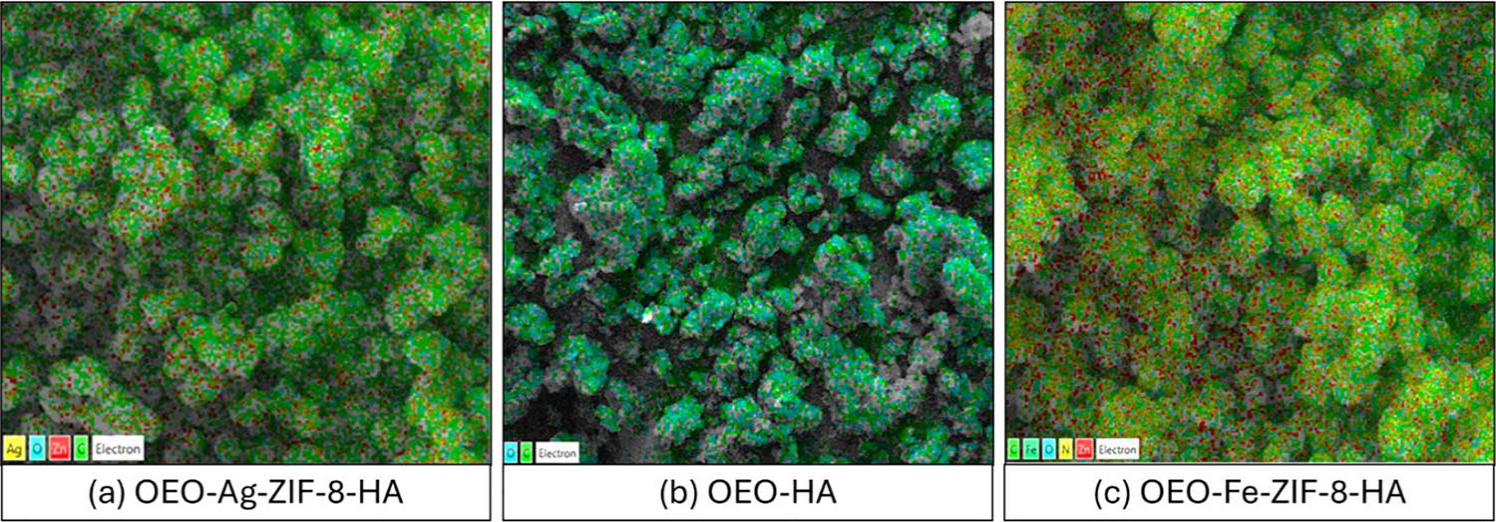
EM of (a) OEO‐Ag‐ZIF‐8‐HA; (b) OEO‐HA; and (c) OEO‐Fe‐ZIF‐8‐HA nanoparticles at magnification levels ranging from 1000 to 5000*x*.

The SEM (Figure 1) and complementary MAP (Figure 2) analyses reveal the distinct morphologies and compositions of OEO–Ag–ZIF‐8–HA, OEO–Fe–ZIF‐8–HA, and OEO–HA nanoparticles. OEO–Ag–ZIF‐8–HA displays uniform polyhedral structures with smooth, well‐defined surfaces (100–200 nm), indicating that Ag^+^ incorporation preserved ZIF‐8 crystallinity and enhanced surface stability (Guo et al. 2024). In contrast, OEO–Fe–ZIF‐8–HA shows rougher, slightly elongated particles with partial agglomeration, attributed to Fe^2^^+^ substitution that distorts Zn–N coordination and slows crystal growth (Nguyen et al. 2025). OEO–HA appears amorphous and densely aggregated due to the absence of metal coordination centers and strong hydrogen bonding between HA and OEO, which reduces porosity (Liu et al. 2019b).

MAP further validates the homogeneous distribution of key elements across all nanoparticle surfaces. OEO–Ag–ZIF‐8–HA exhibits even co‐localization of Ag and Zn with C and O, verifying successful Ag incorporation and HA coating; dispersed Ag suggests effective substitution or surface anchoring, supporting sustained ion release (Li et al. 2013). OEO–Fe–ZIF‐8–HA shows overlapping Fe, Zn, and N signals, confirming Fe^2^^+^ integration within the framework and potential redox synergy (Nguyen et al. 2025). OEO–HA maps reveal only C and O from HA and OEO, confirming its non‐metallic, amorphous nature (Pontes‐Quero et al. 2021). Overall, SEM and mapping results verify successful synthesis, uniform metal incorporation, and effective HA integration across all formulations (Nguyen 2025).

#### 3.1.2 TEM

**FIGURE 3:**
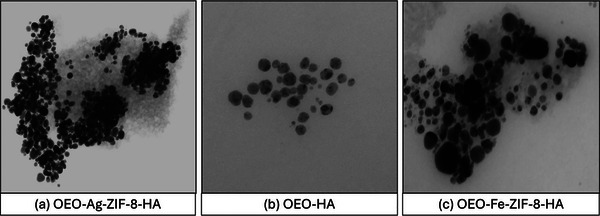
TEM images of (a) OEO‐Ag‐ZIF‐8‐HA; (b) OEO‐HA; and (c) OEO‐Fe‐ZIF‐8‐HA nanoparticles with scale calibrations ranging from 100 – 200 nm.

TEM (Figure 3) revealed clear morphological differences among OEO–Ag–ZIF‐8–HA, OEO–Fe–ZIF‐8–HA, and OEO–HA nanoparticles. OEO–Ag–ZIF‐8–HA exhibited well‐defined polyhedral structures with smooth surfaces and slight agglomeration, indicating that Ag^+^ maintained ZIF‐8 crystallinity but caused minor lattice distortions (Hugenschmidt et al. 2020). The particles were highly dispersed and averaged below 150 nm, reflecting Ag^+^‐induced nucleation that limited crystal growth. OEO–Fe–ZIF‐8–HA showed more irregular, elongated particles with rougher surfaces, consistent with Fe^2^^+^ substitution disrupting crystal symmetry and promoting anisotropic growth. In contrast, OEO–HA formed quasi‐spherical, amorphous aggregates typical of HA matrices encapsulating OEO droplets. All samples exhibited a thin, uniform HA coating that enhanced dispersion, reduced aggregation, and confirmed effective surface modification critical for biocompatibility and colloidal stability (Nguyen 2025).

#### 3.1.3 FTIR

**FIGURE 4:**
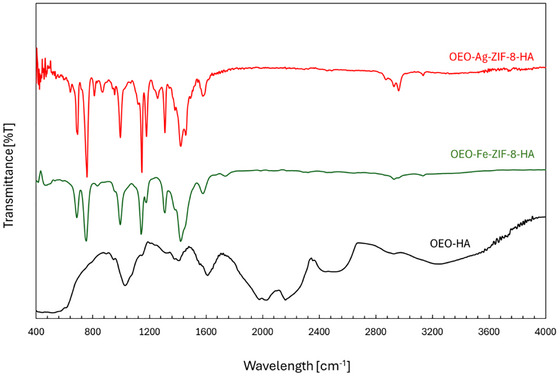
FTIR spectra of OEO‐Fe‐ZIF‐8‐HA, OEO‐Ag‐ZIF‐8‐HA, and OEO‐HA nanoparticles.

FTIR spectra of OEO–Fe–ZIF‐8–HA, OEO–Ag–ZIF‐8–HA, and OEO–HA (Figure 4) verified successful integration of metal‐doped ZIF‐8 frameworks within the OEO–HA matrix. In OEO–HA, the broad band at 3200–3400 cm^−^^1^ corresponds to O─H/N─H stretching, confirming hydrogen bonding between HA and OEO phenolics such as carvacrol and thymol (Lewandowska and Szulic 2021; Nurzyńska‐Wierdak and Walasek‐Janusz 2025). Peaks at 2920 and 2850 cm^−^^1^ indicate C─H stretching of OEO, while absorptions at 1730 (C ═ O), 1650 (amide I), and 1550 cm^−^^1^ (amide II) reflect HA's carboxyl and amide groups (Popescu et al. 2006; Smith 2018). Signals at 1410–1040 cm^−^^1^ are attributed to C─O/C─O─C vibrations of saccharide and phenolic structures (Estrada et al. 2019).

For OEO–Fe–ZIF‐8–HA and OEO–Ag–ZIF‐8–HA, the O–H/N–H band shifted to lower wavenumbers with reduced intensity, indicating coordination between HA hydroxyl/carboxyl groups and metal centers (Mittal et al. 2022). New absorptions near 420–450 cm^−^^1^ correspond to metal–N and metal–O bonds, confirming successful metal incorporation into ZIF frameworks (Wei et al. 2014). OEO–Ag–ZIF‐8–HA showed stronger 1000–1500 cm^−^^1^ bands, reflecting Ag's higher affinity for O/N atoms (Usman and Suliman 2023), whereas Fe doping produced broader, less intense peaks due to partial amorphization. Retained OEO peaks at 2920, 1730, and 1040 cm^−^^1^ confirmed essential oil stability within the matrix (Radeva et al. 2025). Collectively, these spectral features verify strong organic–inorganic bonding and the formation of stable, metal‐doped hybrid nanostructures.

#### 3.1.4 XRD Analysis

**FIGURE 5:**
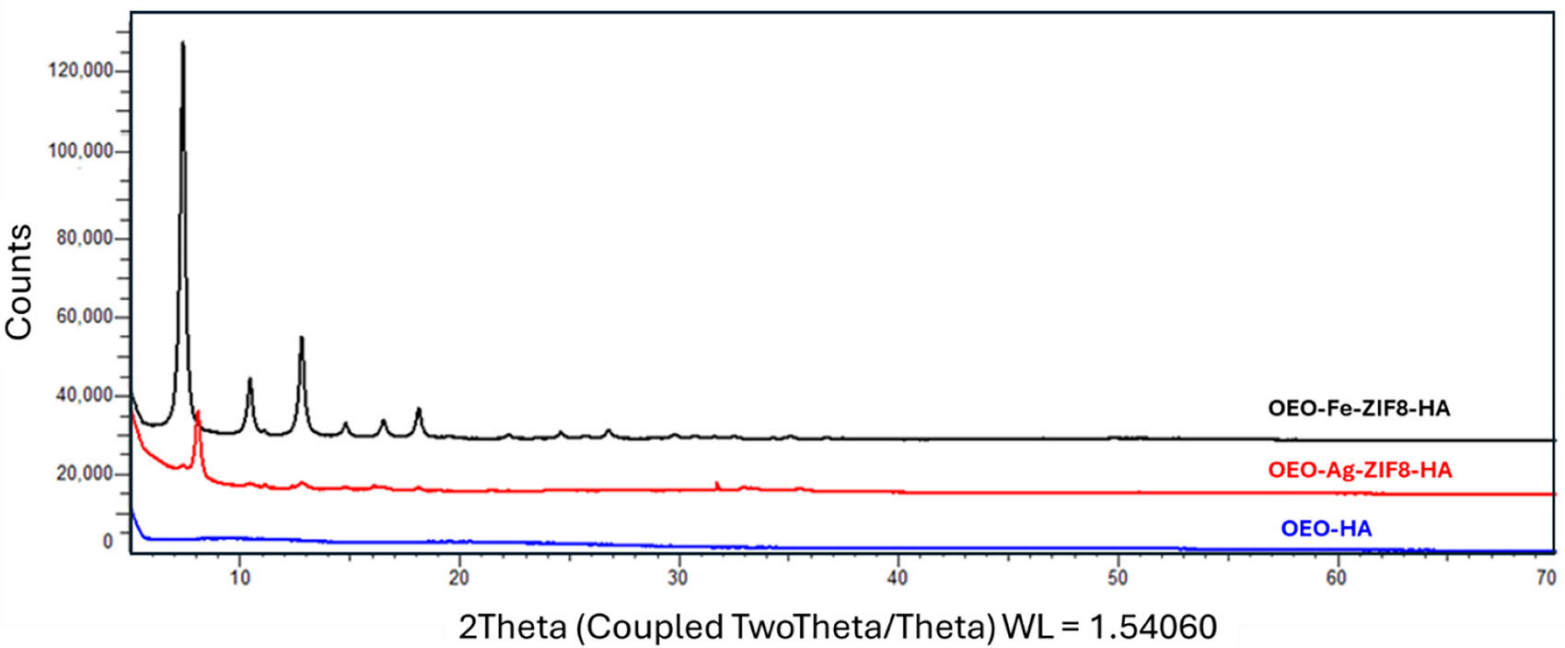
XRD pattern of OEO‐Fe‐ZIF‐8‐HA, OEO‐Ag‐ZIF‐8‐HA, and OEO‐HA nanoparticles.

**FIGURE 6:**
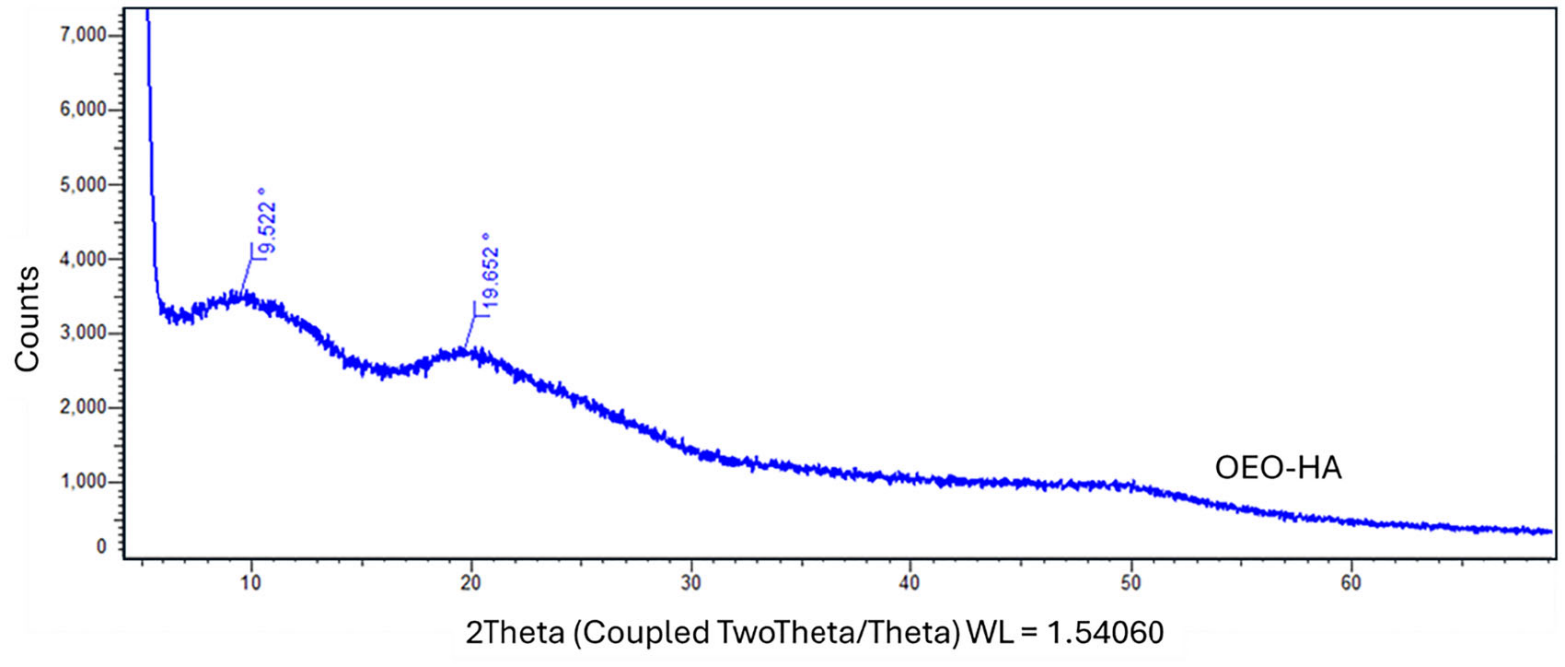
XRD pattern of the OEO‐HA nanoparticle.

The XRD patterns of OEO–Fe–ZIF‐8–HA, OEO–Ag–ZIF‐8–HA, and OEO–HA (Figure 5) clearly differentiate the amorphous OEO–HA matrix from the crystalline metal‐doped composites. OEO–HA shows a broad halo at 2θ = 9–20° (Figure 6), confirming its non‐crystalline polymeric nature, typical of HA, and further disrupted by OEO incorporation, which enhances solubility and dispersion (Radeva et al. 2025).

Both OEO–Fe–ZIF‐8–HA and OEO–Ag–ZIF‐8–HA exhibit sharp reflections at 2θ ≈ 7.3°, 10.4°, 12.7°, 14.7°, 16.4°, 18.0°, 24.5°, 26.7°, 29.6°, and 32.4°, characteristic of ZIF‐8's sodalite‐type structure (Alowasheeir et al. 2024). Compared with pristine ZIF‐8, peak intensities are slightly reduced, suggesting mild lattice distortion or partial amorphization due to metal incorporation. In OEO–Fe–ZIF‐8–HA, broader peaks indicate Fe^2^^+^ substitution for Zn^2^^+^ and minor disruption of long‐range order, while OEO–Ag–ZIF‐8–HA retains sharper reflections, implying Ag^+^ localization on surfaces or within pores rather than lattice sites (Usman and Suliman 2023). The preserved ZIF‐8 diffraction profile in both composites confirms structural integrity despite metal doping (Mittal et al. 2022; Nguyen et al. 2025). XRD results verify successful retention of the crystalline ZIF‐8 framework within the HA–OEO matrix, with Fe doping inducing slight lattice disorder and Ag maintaining higher structural order (Nguyen 2025).

#### 3.1.5 ZP

**FIGURE 7:**
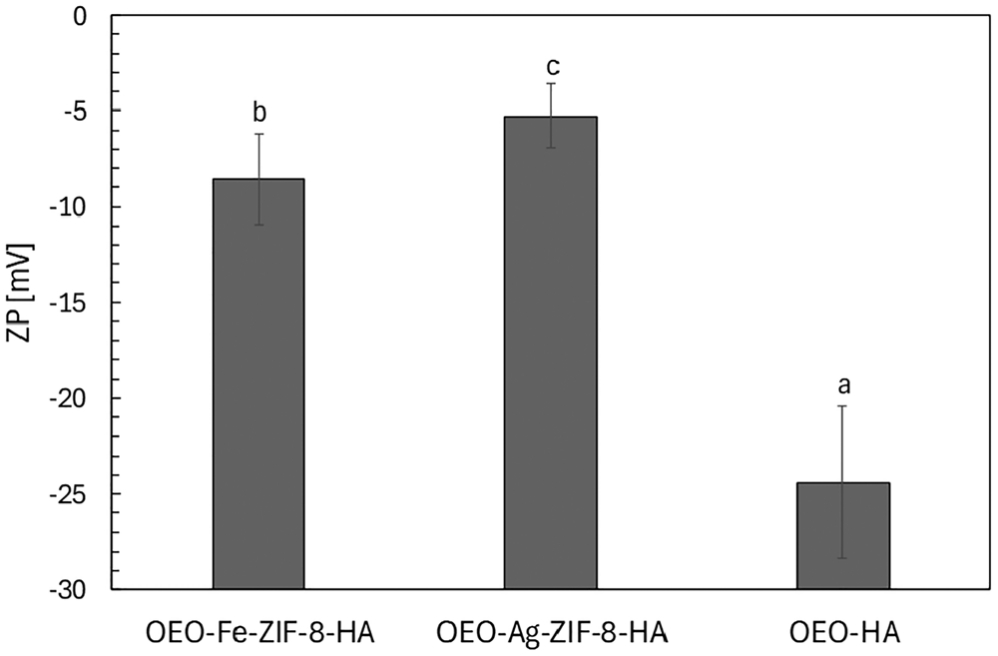
ζ, ZP (mV) of OEO‐Fe‐ZIF‐8‐HA, OEO‐Ag‐ZIF‐8‐HA, and OEO‐HA nanoparticles. ^a,b,c^ Means without a common subscript in the same group are significantly different (*p* < 0.05) from the others. Values are the average of three replications.

ZP (ζ) values of OEO–Fe–ZIF‐8–HA (−10.24 ± 2.36 mV), OEO–Ag–ZIF‐8–HA (−7.13 ± 1.68 mV), and OEO–HA (−22.87 ± 3.99 mV) highlight the effect of metal doping and HA coating on nanoparticle surface charge (Figure 7). ZP, a measure of electrostatic stability and particle–cell interaction, directly influences colloidal behavior and antibacterial activity (Maillard et al. 2021). Typically, particles with ζ beyond ± 20 mV exhibit electrostatic stabilization, while weaker values rely on steric effects (Honary and Zahir 2013; Gumustas et al. 2017). OEO–HA showed the most negative potential (−22.87 mV), consistent with HA's anionic nature and values commonly reported for HA‐coated or essential oil‐based nanocarriers (Alipoor et al. 2022; Zamboni et al. 2022; Neto et al. 2025).

Incorporation of ZIF‐8 cores reduced surface negativity, with Fe‐ and Ag‐doped composites displaying attenuated ζ values (−10.24 and −7.13 mV), like trends observed in HA‐functionalized MOFs (Makhetha et al. 2020; Mahmoudi et al. 2025; Wu et al. 2023). This shift results from partial masking of HA's charge by the neutral or positively coordinated Zn–N, Fe–N, or Ag–N sites of ZIF‐8. The weaker negative charge may enhance bacterial interaction by reducing electrostatic repulsion, thereby improving antimicrobial contact (Franci et al. 2015; Zamboni et al. 2022; Neto et al. 2025). Slightly lower ζ in Fe‐doped systems likely reflects greater surface reactivity and localized charge formation, whereas Ag^+^ interactions with HA carboxylates shift ζ toward neutrality (Kuyukina et al. 2022). Despite limited electrostatic stabilization, HA provides steric hindrance that prevents aggregation.

Overall, ζ measurements reveal that OEO–HA maintains strong electrostatic stability, while metal‐doped ZIF‐8–HA composites balance reduced charge with improved bacterial affinity. These results align with previous reports on HA‐functionalized MOFs and essential oil carriers, demonstrating how dopant chemistry and HA coating jointly govern nanoparticle stability and antimicrobial performance (Nikam et al. 2022; Nguyen 2025).

#### 3.1.6 PS

**FIGURE 8:**
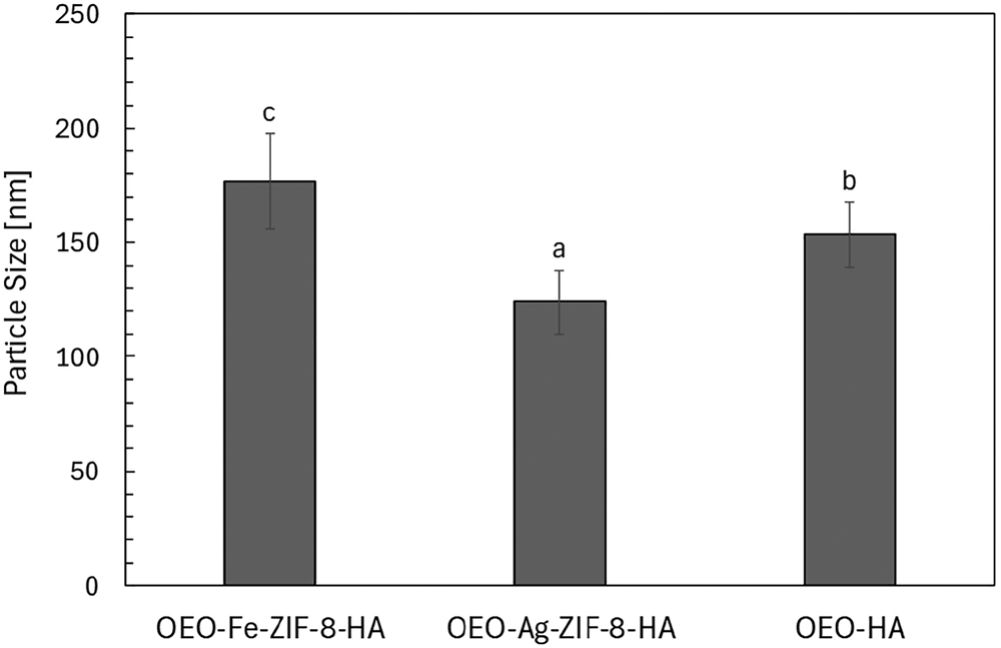
PS (nm) of OEO‐Fe‐ZIF‐8‐HA, OEO‐Ag‐ZIF‐8‐HA, and OEO‐HA nanoparticles. ^a,b,c^ Means without a common subscript in the same group are significantly different (*p* < 0.05) from the others. Values are the average of three replications.

Dynamic light scattering revealed nanoscale PSs for OEO–Ag–ZIF‐8–HA (123.8 nm), OEO–HA (153.4 nm), and OEO–Fe–ZIF‐8–HA (176.9 nm) (Figure 8). These values align with reported ranges for HA‐based nanocarriers and essential‐oil nano‐emulsions (20–200 nm), where small diameters enhance colloidal stability through high surface area and steric hindrance (Rimple and Newton 2018; Qamar et al. 2019; Liu et al. 2019a; Li et al. 2025). HA's hydrophilic, negatively charged surface further improves dispersion stability by forming a hydrated shell. All formulations remain within the optimal 100–200 nm window favorable for biomedical use, ensuring extended suspension stability, EPR‐mediated accumulation, and efficient endocytic uptake (Nakamura et al. 2016).

Minor size variations reflect core composition and HA interaction. The smallest (*p* < 0.05) particles, OEO–Ag–ZIF‐8–HA (123.8 nm), suggest that Ag^+^ incorporation limits MOF crystal growth via additional nucleation sites, whereas OEO–Fe–ZIF‐8–HA (176.9 nm) experiences partial Fe^2^^+^ substitution and HA crosslinking, producing thicker coatings. OEO–HA (153.4 nm) represents a typical HA‐stabilized oil‐in‐water nano‐emulsion, consistent with other biopolymer‐encapsulated essential oils (<150 nm) (Øye et al. 2023). Essential oil encapsulation increases PS due to the added lipophilic volume, as observed in guest‐loaded ZIF‐8 systems (Li et al. 2025). Overall, all OEO–HA formulations exhibited stable PSs (120–180 nm), typical of HA–MOF hybrids, with Ag doping reducing and Fe doping increasing diameters slightly. Their sub‐200 nm range ensures colloidal stability, high surface functionality, and suitability for biomedical or antimicrobial applications (Zhou et al. 2021; Nguyen 2025).

### 3.2 Cytotoxicity Test

**FIGURE 9:**
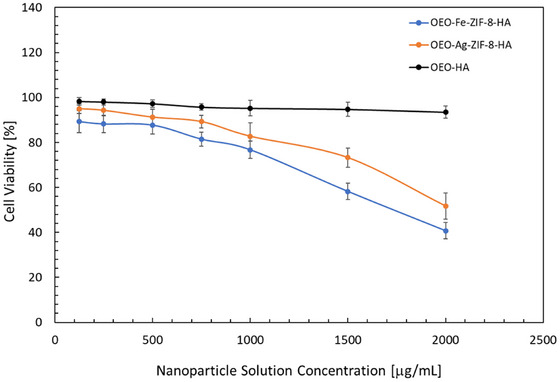
Cytotoxicity of OEO‐Fe‐ZIF‐8‐HA, OEO‐Ag‐ZIF‐8‐HA, and OEO‐HA nanoparticles at different concentrations. Values are the means of three replications.

The cytotoxicity assay (Figure 9) revealed concentration‐dependent differences in biocompatibility among OEO–Ag–ZIF‐8–HA, OEO–Fe–ZIF‐8–HA, and OEO–HA. At 250–500 µg/mL, all formulations maintained high CCK‐8 cell viability (>85–95%), indicating low cytotoxicity under the tested in vitro conditions. OEO–HA showed the highest biocompatibility, sustaining >93% viability even at 2000 µg/mL, consistent with HA's cytoprotective, biocompatible nature (Zhu et al. 2017b; Zamboni et al. 2022). In contrast, OEO–Ag–ZIF‐8–HA and OEO–Fe–ZIF‐8–HA exhibited dose‐dependent toxicity, with significant declines at ≥1000 µg/mL. At 2000 µg/mL, OEO–Ag–ZIF‐8–HA and OEO–Fe–ZIF‐8–HA reduced viability to 51.75% and 40.75%, respectively, with Fe‐doping inducing greater cytotoxicity due to enhanced ROS generation and mitochondrial stress (Luo et al. 2019; Wang et al. 2025). Silver incorporation produced milder toxicity, as Ag^+^ partially stabilizes the ZIF‐8 framework, reducing Zn^2^^+^ ion release and controlling Ag^+^ dissolution while retaining antibacterial potency (Makhetha et al. 2020). Encapsulated OEO also contributed to controlled cytotoxicity, although its phenolic compounds (carvacrol, thymol) are toxic at high free concentrations (>100 µg/mL) but are safely released from HA–ZIF‐8 matrices (Sivropoulou et al. 1996; Chiriac et al. 2021). The HA coating provides a diffusion barrier that mitigates exposure, explaining the high cell viability of OEO–HA across all concentrations. Overall, viability above 85% for Fe‐ and Ag‐doped systems up to 500 µg/mL suggests a broad therapeutic window combining antimicrobial efficacy with low cytotoxicity within the evaluated exposure range.

These findings suggest that OEO–HA is suitable for applications requiring extended exposure or higher concentrations, while OEO–Ag–ZIF‐8–HA and OEO–Fe–ZIF‐8–HA offer a balance between enhanced antibacterial efficacy and acceptable cytocompatibility for short‐term or high‐efficacy antimicrobial applications under controlled conditions (Nguyen 2025).

### 3.3 Assessment of Antibacterial Activity

#### 3.3.1 Disk Diffusion Test

**FIGURE 10:**
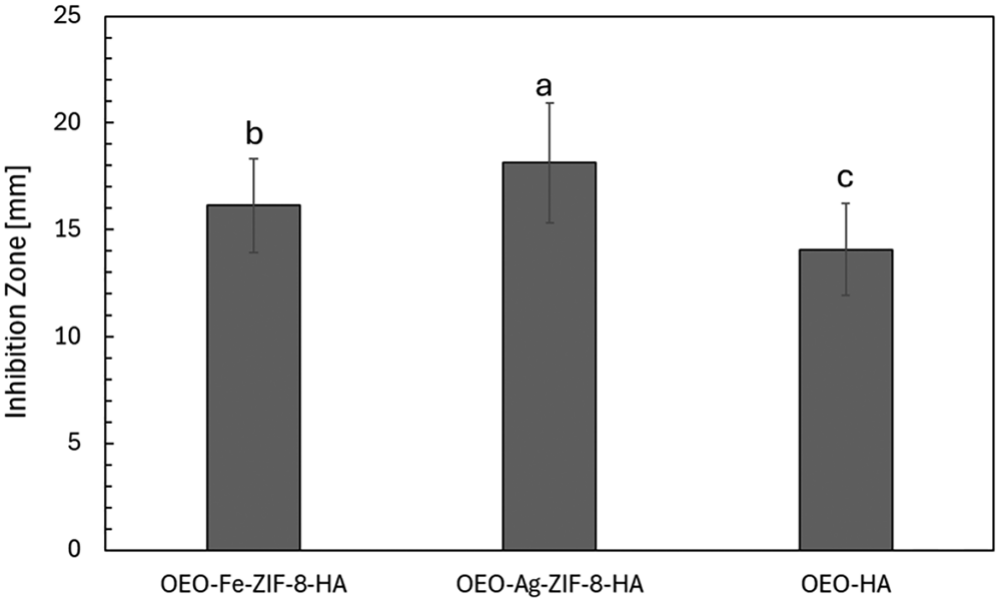
Disk diffusion test results with inhibition zone (mm) of OEO‐Fe‐ZIF‐8‐HA, OEO‐Ag‐ZIF‐8‐HA, and OEO‐HA against *Listeria monocytogenes* cocktail. Values are the means of three replications. ^a,b,c^ Means within a column, which are not followed by a common superscript letter, are significantly different (*p* < 0.05).

The disk diffusion assay (Figure 10), applied as a qualitative indicator of antibacterial diffusion and relative inhibitory potential, revealed apparent (*p* < 0.05) differences in antibacterial activity among OEO–Ag–ZIF‐8–HA, OEO–Fe–ZIF‐8–HA, and OEO–HA nanoparticles against *Listeria monocytogenes*. OEO–Ag–ZIF‐8–HA showed the most potent inhibition (18.13 ± 2.78 mm), followed by OEO–Fe–ZIF‐8–HA (16.13 ± 2.20 mm) and OEO–HA (14.07 ± 2.10 mm) (*p* < 0.05). These results confirm the synergistic enhancement provided by metal doping and ZIF‐8 integration, consistent with reports that hybrid nanostructures combining metals, MOFs, and phytochemicals yield superior antibacterial efficacy (Makhetha et al. 2020).

OEO–HA alone exhibited notable inhibition, consistent with OEO's phenolic constituents, carvacrol, and thymol, which disrupt bacterial membranes and quorum sensing (Kalaba et al. 2024; Moghrovyan and Sahakyan 2024). Its 14 mm zone compares favorably with other polymeric OEO systems, such as alginate nanofibers, which show 10 mm zones (Orisawayi et al. 2025), indicating efficient OEO release. Metal incorporation significantly improved activity, with Ag >Fe, aligning with previous studies with Ag‐based hybrids that achieve 16–19 mm inhibition zones against *Listeria* (Gudkov et al. 2021; Dove et al. 2023; Al‐Shemy et al. 2025). Synergy between OEO and Ag^+^ accelerates bacterial killing with OEO compromising membranes, enhancing silver ion uptake and toxicity (Scandorieiro et al. 2022).

ZIF‐8 frameworks further contribute via controlled Zn^2^^+^ release and physical disruption (Di Matteo et al. 2023; Tho et al. 2025), while HA provides colloidal stability and sustained delivery (Alipoor et al. 2022). Together, these components act synergistically, including OEO damages membranes, Ag and Fe generate oxidative stress, ZIF‐8 releases Zn^2^^+^, and HA stabilizes the system, yielding the relative ranking of formulations Ag > Fe > non‐metal doping. Such multi‐target mechanisms enhance antimicrobial potency while minimizing resistance development (Yousefi et al. 2020; Wang et al. 2023). In conclusion, the high inhibition zones achieved by OEO–Ag–ZIF‐8–HA highlight how coupling botanical antimicrobials with inorganic nanomaterials produces robust, broad‐spectrum antibacterial performance, guiding subsequent quantitative MIC and an MBC analyses in the next section (Nguyen 2025).

#### 3.3.2 MIC and MBC

The MIC and MBC results (Table 1) revealed that OEO–Ag–ZIF‐8–HA exhibited the most potent antibacterial activity against *Listeria monocytogenes*, with an MIC of 125 µg/mL and MBC of 375 µg/mL, significantly lower (*p* < 0.05) than OEO–Fe–ZIF‐8–HA (875/2625 µg/mL) and OEO–HA (1250/4375 µg/mL). This tenfold improvement over Fe‐doped and non‐metal formulations demonstrates that Ag^+^ incorporation significantly enhances antibacterial potency (*p* < 0.05). Silver's intense bactericidal action, reported with MICs as low as 6.2 µg/mL (Huq 2025), outperforms Fe^2^^+^ systems, which rely on weaker Fenton‐mediated ROS generation (Luo et al. 2019; Wang et al. 2025). All three composites showed MBC/MIC ratios of 3–3.5, below the ≤4 threshold for bactericidal activity (Makade et al. 2024), confirming lethal effects rather than mere inhibition.

**TABLE 1:** MIC and MBC of tested nanoparticle solutions against *Listeria monocytogenes* cocktail after 24 h exposure. ^a,b,c^ Means without a common subscript in the same column are significantly different (*p* < 0.05) from the others.

Nanoparticle solution	MIC value (µg/mL)	MBC value (µg/mL)	MBC/MIC ratio
OEO‐Fe‐ZIF‐8‐HA	875^b^	2625^b^	3
OEO‐Ag‐ZIF‐8‐HA	125^a^	375^a^	3
OEO‐HA	1250^c^	4375^c^	3.5

*Note*: ^a,b,c^ Means without a common subscript in the same column are significantly different (*p* < 0.05) from the others.

The superior efficacy of OEO–Ag–ZIF‐8–HA arises from synergistic antibacterial mechanisms. Encapsulated OEO provides membrane‐active compounds that inhibit cell envelopes and metabolism (Guo et al. 2024), while Ag^+^ ions contribute to enzyme inactivation, DNA damage, and oxidative stress (Fan et al. 2018). The ZIF‐8 framework further releases Zn^2^^+^, increasing membrane permeability and ROS generation (Meng et al. 2022). In contrast, OEO–HA's higher (*p* < 0.05) MIC (1250 µg/mL) reflects reliance on slower OEO release and the absence of metallic antibacterial activity (Granata et al. 2018). Fe doping modestly improved efficacy (MIC 875 µg/mL) via limited oxidative catalysis, though less potent than Ag^+^.

Given *L. monocytogenes*’ role as a leading cause of fatal foodborne illness (Anupama et al. 2025; De Souza et al. 2025), these results underscore the critical value of Ag‐based hybrids capable of total bacterial elimination at low concentrations. The broad‐spectrum nature of OEO and Ag^+^ suggests wider applicability, as AgNPs display comparable or greater efficacy against Gram‐negative pathogens such as *E. coli* O157:H7, *Salmonella Typhimurium*, and *Vibrio parahaemolyticus* (Zarei et al. 2013). Likewise, OEO inhibits *Staphylococcus aureus* and *E. coli*, though higher doses are needed for Gram‐negative bacteria (Schneider et al. 2023). Overall, the exceptionally low MIC/MBC values of OEO–Ag–ZIF‐8–HA highlight the strong synergistic bactericidal performance of essential oils, silver, and Zn‐based frameworks as an effective strategy for controlling *L. monocytogenes* and other foodborne pathogens.

### 3.4 Nanoparticle Approaches to Inhibit *Listeria Monocytogenes* Biofilms

#### 3.4.1 Latex Surface Characterization

**FIGURE 11:**
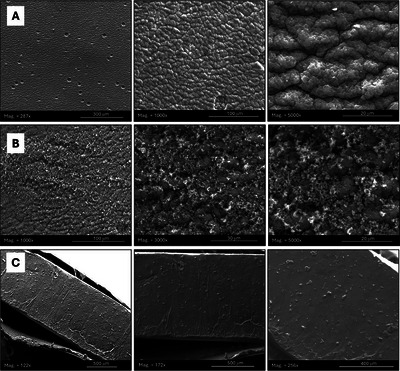
SEM images of latex surfaces with (A) top view of the surface; (B) top view of the surface with OEO‐Ag‐ZIF‐8‐HA nanoparticles attached; and (C) side view of the coupons.

The SEM micrographs (Figure 11) revealed distinct morphological changes on latex surfaces after OEO–Ag–ZIF‐8–HA deposition. The pristine latex (Figure 11A) displayed a smooth, slightly grooved texture typical of fused polymeric particles, offering limited but hydrophobic sites for bacterial attachment. After coating, granular aggregates of OEO–Ag–ZIF‐8–HA were distributed (Figure 11B), confirming successful surface immobilization. The side‐view image (Figure 11C) showed a continuous, thin nanoparticle layer adhering to the surface without substrate penetration, ensuring structural integrity and maximum antibacterial exposure. These observations demonstrate that nanoparticle deposition transformed the latex from smooth to nano‐textured, enhancing surface roughness and potential antibacterial contact efficiency.

**FIGURE 12:**
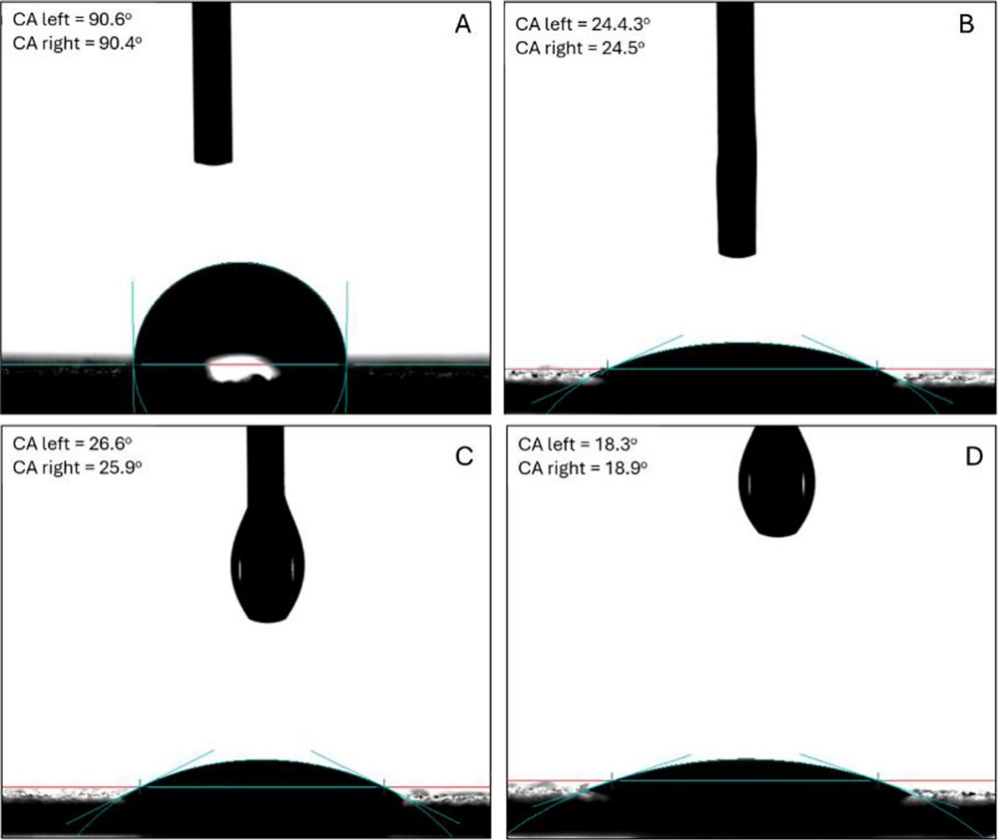
Digital images of (A) water; (B) OEO‐Fe‐ZIF‐8‐HA; (C) OEO‐Ag‐ZIF‐8‐HA; and (D) OEO‐HA nanoparticle solutions on latex surfaces.

Contact angle analysis (Figure 12, Table 2) further confirmed improved wettability. Untreated latex exhibited hydrophobicity (91.62 ± 8.96°) (Yuan and Lee 2013), while treatment with OEO–Fe–ZIF‐8–HA, OEO–Ag–ZIF‐8–HA, and OEO–HA reduced angles to 27.25 ± 6.51°, 28.51 ± 2.13°, and 20.61 ± 3.53°, respectively. The decrease reflects enhanced hydrophilicity from hydroxyl, carboxyl, and amide groups in HA and OEO (Pan et al. 2011; Tongnuanchan and Benjakul 2014). Slightly higher angles for Fe‐ and Ag‐doped formulations stem from partial aggregation or nonuniform coverage. These more hydrophilic surfaces improve coating uniformity and can reduce bacterial adhesion (Li et al. 2021; Su et al. 2025), facilitating better antimicrobial contact through increased surface energy (Steinerová et al. 2022; Sevimli‐Yurttas et al. 2024).

**TABLE 2:** Contact angle of water, OEO‐Fe‐ZIF‐8‐HA, OEO‐Ag‐ZIF‐8‐HA, and OEO‐HA nanoparticle solutions on latex surfaces. Values are the means of three replications. ^a,b,c^ Means within a column, which are not followed by a common superscript letter, are significantly different (*p* < 0.05).

Treatment	Contact angle (o)
Water	91.62 ± 8.96^c^
OEO‐Fe‐ZIF‐8‐HA	27.25 ± 6.51^b^
OEO‐Ag‐ZIF‐8‐HA	28.51 ± 2.13^b^
OEO‐HA	20.61 ± 3.53^a^

*Note*: ^a,b,c^ Means without a common subscript in the same column are significantly different (*p* < 0.05) from the others.

**FIGURE 13:**
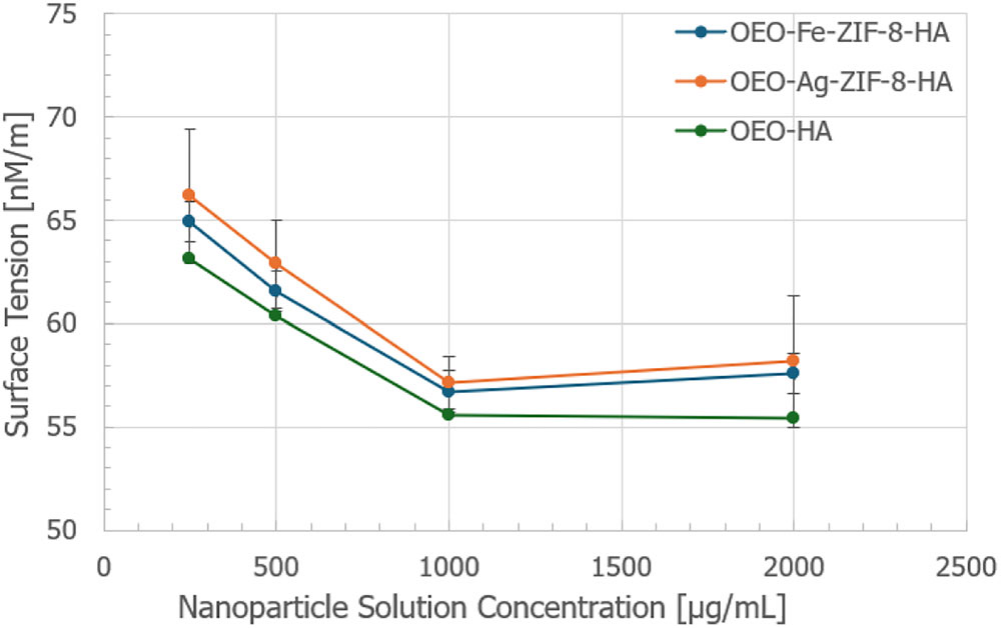
Surface tension changes with different nanoparticle solution concentrations of OEO‐Fe‐ZIF‐8‐HA, OEO‐Ag‐ZIF‐8‐HA, and OEO‐HA.

Surface tension results (Figure 13) supported these findings with latex surfaces treated with OEO‐based nanocomposites exhibited reduced interfacial energy compared to water (72.3 mN/m at 25°C). Increasing nanoparticle concentration from 250 to 1000 µg/mL decreased γ, followed by a slight rise at 2000 µg/mL due to particle aggregation (Karademir and Özkan 2025). OEO–HA showed the lowest tension (∼55.5 mN/m at 1000 µg/mL), correlating with its highest hydrophilicity and smallest contact angle. The hydrophilic HA chains and amphiphilic OEO enhanced hydrogen bonding and spreading (Tao et al. 2025). Fe‐ and Ag‐doped ZIF‐8 hybrids also lowered surface tension (56–57 mN/m) through polar metal sites and surface roughness, though slightly less than OEO–HA due to restricted HA flexibility (Kuznetsov et al. 2021). The mild increase at higher doses reflects interparticle crowding (Chai et al. 2020).

These results demonstrate that OEO–HA and its metal‐doped derivatives significantly enhance the hydrophilicity and interfacial properties of latex, transforming it from hydrophobic to bioactive, hydrophilic, and suitable for antibacterial and coating applications.

##### 3.4.1.1 Effect of Nanoparticles on *Listeria Monocytogenes* on Latex Surfaces

Table 3 shows that OEO–Ag–ZIF‐8–HA and OEO–HA nanoparticles significantly (*p* < 0.05) inhibited *Listeria monocytogenes* adhesion and survival on latex surfaces, confirming their strong antimicrobial potential for glove‐type materials and food packaging. Untreated latex (7.62 ± 0.27 log CFU/cm^2^) and the solvent control (7.32 ± 0.26 log CFU/cm^2^) supported heavy colonization, consistent with latex's hydrophobic, nutrient‐retentive properties that promote *Listeria* adhesion and biofilm development (Lee et al. 2019; Ban‐Cucerzan et al. 2025).

**TABLE 3:** Adhesion and growth inhibition of *Listeria monocytogenes* on latex surfaces in the presence of OEO‐Ag‐ZIF‐8‐HA and OEO‐HA nanoparticles. Values are the means of three replications. ^a,b,c,d,e,f^ Means within a column, which are not followed by a common superscript letter, are significantly different (*p* < 0.05).

Treatment	Log CFU/cm^2^
Control	7.62 ± 0.27^f^
PBS + 5% MeOH	7.32 ± 0.26^f^
MIC of OEO‐Ag‐ZIF‐8‐HA	5.72 ± 0.37^de^
MBC of OEO‐Ag‐ZIF‐8‐HA	4.01 ± 0.38^c^
2xMBC of OEO‐Ag‐ZIF‐8‐HA	0.64 ± 0.29^a^
MIC of OEO‐HA	6.19 ± 0.37e
MBC of OEO‐HA	5.23 ± 0.38^d^
2xMBC of OEO‐HA	3.22 ± 0.33^b^

*Note*: ^a,b,c,d,e,f^ Means without a common subscript in the same column are significantly different (*p* < 0.05) from the others.

Both nanoparticle treatments produced concentration‐dependent reductions. At the MIC, OEO–Ag–ZIF‐8–HA and OEO–HA reduced counts to 5.72 ± 0.37 and 6.19 ± 0.37 log CFU/cm^2^, respectively (*p* < 0.05), corresponding to ∼2 log reductions. OEO–Ag–ZIF‐8–HA showed greater efficacy due to synergistic activity between Ag^+^ ions and OEO phenolics, which disrupt cell membranes, proton gradients, and protein function (Yin et al. 2020; Scandorieiro et al. 2022). The ZIF‐8 matrix provides sustained Ag^+^ release and alleviation of oxidative stress, while the HA coating ensures nanoparticle stability, biocompatibility, and strong surface adhesion (Schubert and Chanana 2019; Mohammed et al. 2022; Zheng et al. 2024).

At the MBC, viable counts significantly (*p* < 0.05) fell to 4.01 ± 0.38 log CFU/cm^2^ for OEO–Ag–ZIF‐8–HA and 5.23 ± 0.38 for OEO–HA, while 2×MBC achieved near‐complete eradication (0.64 ± 0.29 and 3.22 ± 0.33 log CFU/cm^2^, respectively). This dose‐dependent response aligns with typical MOF‐based nanomaterial behavior, combining physical disruption and chemical lethality (Cuthbert et al. 2022). The OEO–Ag–ZIF‐8–HA mechanism integrates Ag^+^ oxidative damage with OEO‐driven membrane rupture, whereas OEO–HA relies mainly on diffusion‐based inhibition.

The antibacterial hierarchy (2×MBC > MBC > MIC > control) highlights both concentration dependence and formulation specificity. Ag^+^ doping intensifies antimicrobial potency and stabilizes OEO against oxidation and volatilization (Thapliyal et al. 2025). Meanwhile, HA enhances nanoparticle adhesion to latex via hydrogel bonding and van der Waals interactions (Mohammed et al. 2022; Yoon et al. 2025). Overall, OEO–Ag–ZIF‐8–HA achieved the most pronounced biofilm reduction on latex, demonstrating a synergistic mechanism among Ag^+^, OEO, and HA that addresses persistent contamination challenges in food‐processing environments (Nguyen 2025).

##### 3.4.1.2 Effect of OEO‐Ag‐ZIF‐8‐HA and OEO‐HA on the Removal of the Established *Listeria Monocytogenes* Biofilms on Latex Surfaces

Table 4 highlights the significant (*p* < 0.05) differences in the antibiofilm efficacy of OEO–Ag–ZIF‐8–HA and OEO–HA nanoparticles on latex surfaces under varying exposure times and mechanical conditions. Latex's hydrophobic, elastic structure promotes *Listeria monocytogenes* adhesion and EPS retention, making biofilm removal challenging (Da Silveira et al. 2009). Baseline OEO–Ag–ZIF‐8–HA treatments (20 × MBC for 1 h or Beads for 1 h) achieved modest (*p* < 0.05) reductions of 1.44 ± 0.37 and 1.18 ± 0.28 log CFU/cm^2^, respectively. Combining both treatments (20×MBC + Beads, 1 h) improved removal to 2.43 ± 0.34 log CFU/cm^2^ (*p* < 0.05), demonstrating that mechanical abrasion enhances nanoparticle penetration and antimicrobial contact (Lin et al. 2021). Prolonged exposure further increased efficacy (*p* < 0.05), with 20 × MBC for 24 h yielding a 3.86 ± 0.41 log CFU/cm^2^ reduction (>99.9%), meeting bactericidal sanitation standards (CLSI 1999).

**TABLE 4:** Log‐reduction (CFU/cm^2^) of *L. monocytogenes* adhered to latex surfaces achieved with different treatments. Values are the means of three replications. ^a,b,c,d,e^ Means within a column, which are not followed by a common superscript letter, are significantly different (*p* < 0.05).

Treatment	Log CFU/cm^2^
20xMBC – 1 h (OEO‐Ag‐ZIF‐8‐HA)	1.44 ± 0.37^b^
Beads – 1h	1.18 ± 0.28^a,b^
20xMBC—Beads – 1 h (OEO‐Ag‐ZIF‐8‐HA)	2.43 ± 0.34^c^
20xMBC – 24 h (OEO‐Ag‐ZIF‐8‐HA)	3.86 ± 0.41^e^
20xMBC – 1 h (OEO‐HA)	1.02 ± 0.25^a^
20xMBC—Beads – 1 h (OEO‐HA)	2.04 ± 0.31^b,c^
20xMBC – 24 h (OEO‐HA)	3.37 ± 0.44^d^

*Note*: ^a,b,c,d,e,f^ Means without a common subscript in the same column are significantly different (*p* < 0.05) from the others.

OEO–HA showed a similar but weaker (*p* < 0.05) trend, achieving 1.02 ± 0.25 log reduction (20 × MBC, 1 h), 2.04 ± 0.31 with agitation, and 3.37 ± 0.44 after 24 h. The absence of Ag^+^ limited oxidative stress, so inhibition relied on OEO phenolics (carvacrol, thymol) disrupting bacterial membranes and proton gradients (Ultee et al. 2002; Kachur and Suntres 2020). Bead agitation helped dismantle EPS layers, improving penetration (Inkyo et al. 2006). Although the reductions were slightly lower than those achieved by traditional sanitizers (Mendez et al. 2022), these nanoparticles acted on more complex latex substrates via sustained release rather than short‐term chemical exposure.

In summary, OEO–Ag–ZIF‐8–HA demonstrated superior biofilm removal through synergistic chemical–mechanical–temporal effects, integrating Ag‐mediated oxidative stress, OEO‐driven membrane disruption, and HA‐facilitated nanoparticle adhesion. The comparable long‐term efficacy to conventional sanitizers, combined with enhanced safety and reusability, positions this nanocomposite as a promising alternative for *L. monocytogenes* control on polymeric and latex food‐contact materials (Abo‐zeid et al. 2022; Singh et al. 2023; Ding et al. 2024).

### 3.5 Applications of Nanoparticle Solutions to Baby Arugula Leaves

#### 3.5.1 Growth Inhibition of *L. Monocytogenes* on the Surfaces of Leaves

**FIGURE 14:**
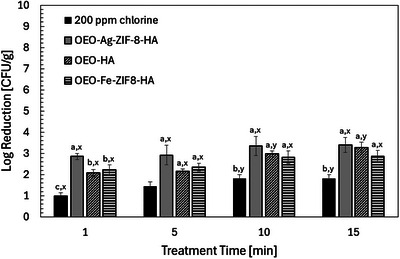
Effect of treatment on the growth inhibition of *L. monocytogenes* attached to the surface of baby arugula leaves. Values are the means of three replications. ^a,b,c^ Means within different times, which are not followed by a common superscript letter, are significantly different within the same time (*p* < 0.05). ^x,y^ Means within a time, which are not followed by a common superscript letter, are significantly different within the same treatment across various time points (*p* < 0.05).

Dipping the leafy green in the nanoparticle solutions demonstrated that all natural nanoparticle systems outperformed the conventional 200 ppm chlorine (*p* < 0.05), confirming their potential as safer and more sustainable post‐harvest sanitizers (Figure 14).

After 1 min of dipping, OEO–Ag–ZIF‐8–HA achieved the highest reduction in *L. monocytogenes* (2.85 ± 0.15 log CFU/g), surpassing OEO–Fe–ZIF‐8–HA (2.22 ± 0.23) and OEO–HA (2.07 ± 0.17), while chlorine achieved only 0.98 ± 0.17 (*p* < 0.05). The strong performance of OEO–Ag–ZIF‐8–HA stems from synergistic effects of Ag^+^ and OEO compounds, which induce oxidative stress, disrupt membranes, and denature key enzymes (Yin et al. 2020; Sonker et al. 2023). Its efficacy increased over time, reaching 3.39 ± 0.35 log reduction at 15 min, reflecting sustained ion release and reactive MOF surface properties (Chen et al. 2024).

OEO–Fe–ZIF‐8–HA exhibited moderate yet consistent activity (2.86 ± 0.29 at 15 min), primarily via Fenton‐like Fe^2^^+^ reactions generating hydroxyl radicals that damage cell walls and DNA (Nguyen et al. 2025). Though less potent than Ag‐based systems, Fe‐doping offers greater biocompatibility and reduced oxidative damage, advantageous for repeated produce washing.

The metal‐free OEO–HA displayed a clear time‐dependent response, increasing from 2.07 ± 0.17 to 3.28 ± 0.26 log reduction between 1–15 min (*p* < 0.05). Its antimicrobial effect derives from stabilized OEO phenolics that penetrate and disrupt bacterial membranes without toxic residues (Omonijo et al. 2018). As a fully biodegradable, food‐grade formulation, OEO–HA is ideal for rinsing delicate produce such as arugula (Martins et al. 2020). By contrast, chlorine achieved only 1.82 ± 0.17 log reduction at 15 min, consistent with *L. monocytogenes* resistance to hypochlorous acid in biofilms (Yang et al. 2024). The 10‐min plateau across treatments suggests surviving cells in leaf crevices and stomata (Yaron and Römling 2014).

Both OEO–Ag–ZIF‐8–HA and OEO–HA significantly outperformed chlorine, with their mechanisms involving ZIF‐8–mediated OEO release, HA‐enhanced dispersion, and metal‐ or OEO‐induced oxidative damage (Jamali et al. 2018; Chen et al. 2024; Rajizadeh and Pourbabaki 2024). While OEO–Ag–ZIF‐8–HA delivered the strongest bactericidal activity, OEO–HA combined high efficacy with excellent biocompatibility (>90% fibroblast viability) (Asensio et al. 2020; Vehapi et al. 2020). The overall hierarchy (OEO–Ag–ZIF‐8–HA > OEO–HA ≈ OEO–Fe–ZIF‐8–HA ≫ chlorine) demonstrates that HA–OEO nanocarriers provide effective, chlorine‐free, biodegradable alternatives for sanitizing minimally processed leafy greens, supporting sustainable and clean‐label food safety practices.

#### 3.5.2 Baby Arugula Leaves’ Quality Features

##### 3.5.2.1 Visual Appearance

**FIGURE 15:**
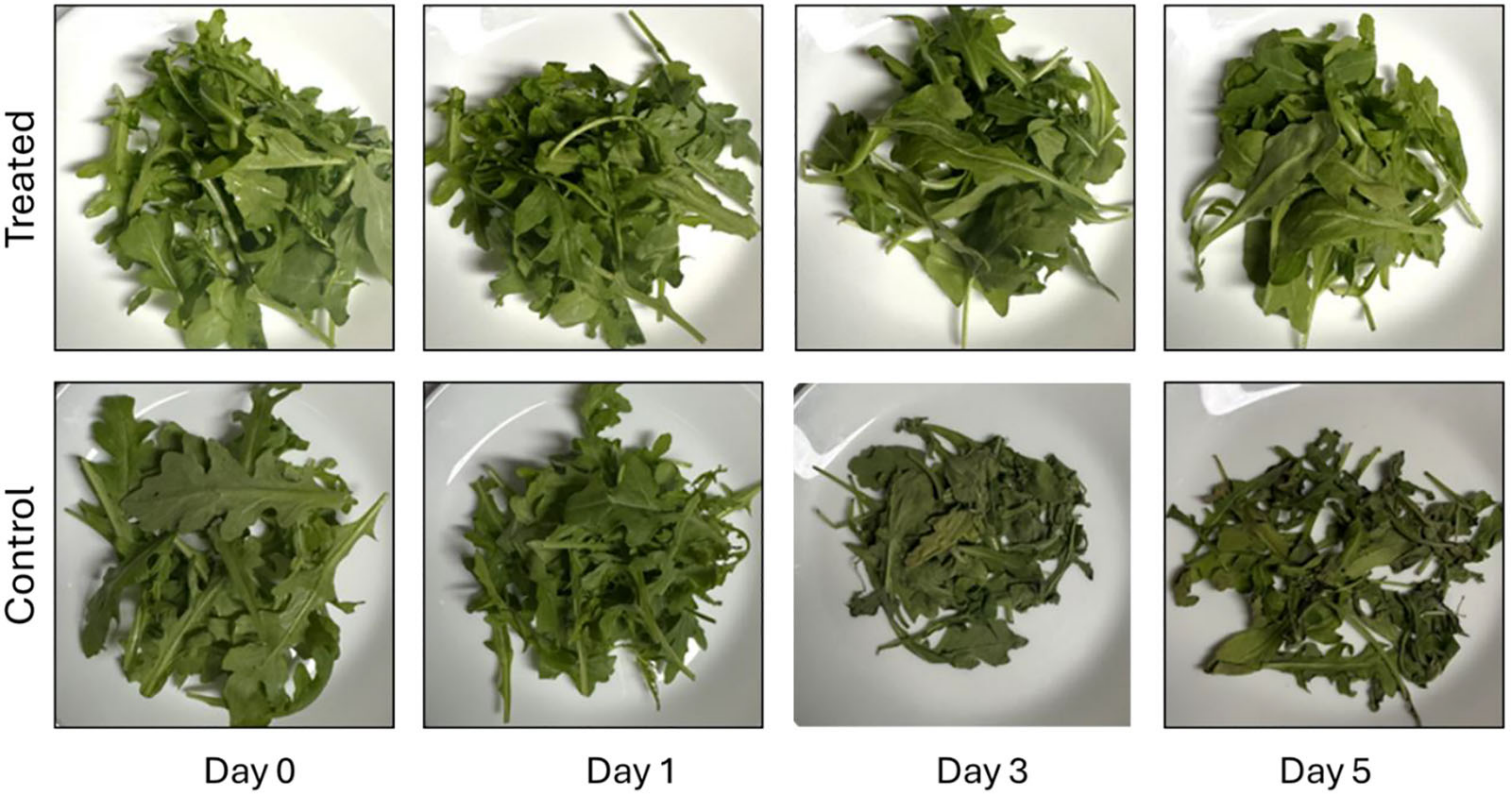
The visual comparison of (top) OEO‐HA‐treated and (bottom) non‐treated (control) samples at day 0, 1, 3, and 5.

The visual appearance of baby arugula leaves treated with OEO‐HA nanoparticles remained consistently fresh and vibrant during storage, showing minimal alterations in color, texture, or overall quality over the five‐day observation period (Figure 15). The OEO‐HA coating created a thin, biocompatible protective layer on the leaf surface, aiding in moisture retention and postponing senescence by restricting oxidative reactions and enzymatic degradation (Rodriguez‐Garcia et al. 2015).

The treated leaves maintained their distinctive bright green color and turgidity, but the untreated leaves displayed gradual visual decline starting on day 3, marked by evident wilting, surface dullness, and moderate yellowing at the edges (Al‐Sulivany et al. 2024). By day 5, the control samples exhibited significant discoloration and compromised structural integrity, indicative of chlorophyll degradation and tissue deterioration linked to moisture loss during storage (Zhu et al. 2017a). The OEO‐HA treatment effectively maintained the visual quality of baby arugula leaves, demonstrating its significant potential as a natural, non‐toxic preservative coating to prolong the postharvest shelf life of fresh leafy greens.

##### 3.5.2.2 Color Changes

**FIGURE 16:**
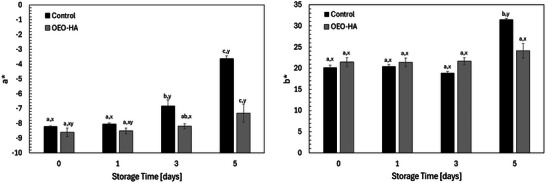
Effect of treatment on the color a* and b* values of baby arugula leaves stored for 15 days at 21°C. Values are the means of three replications. The control samples were baby arugula leaves without any treatment. ^a,b,c^ Means within different times, which are not followed by a common superscript letter, are significantly different within the same time (*p* < 0.05). ^x,y^ Means within a time, which are not followed by a common superscript letter, are significantly different within the same treatment across various time points (*p* < 0.05).

During five days of storage, the control and OEO–HA–treated arugula leaves exhibited distinct color trends in their a* (green–red) and b* (blue–yellow) coordinates, reflecting differences in pigment retention (Figure 16). Both groups began with strongly negative a* values (around –8), typical of fresh green leaves. The control rapidly lost greenness, with a* increasing from –8.22 to –3.63 by day 5, indicating chlorophyll degradation, whereas OEO–HA–treated leaves changed only from –8.61 to –7.31 (*p* < 0.05), maintaining a greener appearance. This slower shift supports the protective role of bioactive coatings in delaying oxidative pigment breakdown.

For the b* coordinate, both treatments showed progressive yellowing, but to different extents: the control increased from 20.16 to 31.50, while OEO–HA rose only to 24.10, indicating reduced carotenoid accumulation and delayed senescence (*p* < 0.05). Similar effects have been reported for edible coatings enriched with essential oils or antioxidants that inhibit chlorophyllase and peroxidase, with key enzymes in chlorophyll degradation (Rehman et al. 2020).

The superior (*p* < 0.05) color retentions of OEO–HA–treated leaves likely stem from the combined actions of HA and OEO. HA forms a semi‐permeable, hydrophilic film that limits gas exchange and moisture loss, preserving turgor and reducing oxidative stress (Chen et al. 2013). Concurrently, OEO's phenolics (carvacrol, thymol) provide antioxidant and antimicrobial protection, curbing enzymatic oxidation and microbial deterioration (Rodriguez‐Garcia et al. 2013). Together, they form a stabilizing barrier that retards pigment loss and senescence. Overall, OEO–HA treatment effectively maintained the natural green color of arugula, supporting its potential as a safe, natural preservation coating that prolongs shelf life and enhances postharvest visual quality.

This study focused on evaluating antimicrobial performance and application efficacy rather than elucidating mechanistic pathways. While enhanced antibacterial activity was observed for OEO–ZIF‐8–HA formulations, specific molecular mechanisms (e.g., membrane disruption, ROS generation, or metal‐ion release kinetics) were not directly investigated and therefore should not be interpreted as confirmed modes of action. Future studies incorporating mechanistic assays such as ROS quantification, membrane integrity analysis, and ion release profiling are needed to clarify the dominant antibacterial mechanisms.

## 4 Conclusion

This study demonstrated that multifunctional nanocomposites integrating OEO, HA, and metal‐doped ZIF‐8 effectively inhibited *Listeria monocytogenes* adhesion and biofilm formation on latex food‐contact surfaces while preserving the microbial and visual quality of baby arugula (*Eruca sativa*) leaves.

Among all formulations, OEO–Ag–ZIF‐8–HA exhibited the most potent antibacterial activity, achieving the lowest MIC/MBC values (125/375 µg mL^−^^1^) and over 5‐log reductions of *L. monocytogenes* on latex at twice‐MBC concentrations, owing to synergistic actions of silver ions, Zn^2^^+^ release from the ZIF‐8 matrix, and phenolic compounds from OEO that together disrupted bacterial membranes and induced oxidative stress.

OEO–Fe–ZIF‐8–HA also displayed significant inhibitory effects but lower potency, while OEO–HA, though metal‐free, maintained strong antimicrobial activity with excellent biocompatibility and no cytotoxicity up to 2000 µg mL^−^^1^. Physicochemical characterization confirmed stable nanoscale structures (120–180 nm) with uniform HA coatings that enhanced colloidal stability and surface adhesion, while SEM, TEM, and EDS mapping revealed homogeneous metal dispersion within the frameworks.

When applied as a rinse on baby arugula leaves, these nanoparticles, particularly OEO–Ag–ZIF‐8–HA and OEO–HA, produced 2–3 log CFU g^−^^1^ reductions within 1–10 min, outperforming 200 ppm chlorine while maintaining leaf freshness, color, and turgidity throughout five‐day storage.

These findings collectively indicate that OEO‐based, HA‐functionalized, and metal‐doped ZIF‐8 nanoparticles are promising chlorine‐free antimicrobial nano‐bioparticles for mitigating *Listeria monocytogenes* contamination on food‐contact surfaces and fresh leafy greens, while preserving product quality under the assessed conditions.

## Author Contributions

**Huy Loc Nguyen**: investigation, writing – original draft, validation, methodology, writing – review and editing, formal analysis, conceptualization. **Rosana G. Moreira**: conceptualization, investigation, writing – original draft, methodology, validation, visualization, writing – review and editing, formal analysis, data curation, supervision, resources. **M. Elena Castell‐perez**: conceptualization, investigation, funding acquisition, writing – original draft, methodology, validation, writing – review and editing, formal analysis, supervision, resources.

## Conflicts of Interest

The authors declare no conflicts of interest.
